# Prediction of population behavior of *Listeria monocytogenes* in food using machine learning and a microbial growth and survival database

**DOI:** 10.1038/s41598-021-90164-z

**Published:** 2021-05-19

**Authors:** Satoko Hiura, Shige Koseki, Kento Koyama

**Affiliations:** Graduate School of Agricultural Science, Hokkaido University, Kita-9, Nishi-9, Kita-ku, Sapporo, 060-8589 Japan

**Keywords:** Applied mathematics, Food microbiology, Data mining

## Abstract

In predictive microbiology, statistical models are employed to predict bacterial population behavior in food using environmental factors such as temperature, pH, and water activity. As the amount and complexity of data increase, handling all data with high-dimensional variables becomes a difficult task. We propose a data mining approach to predict bacterial behavior using a database of microbial responses to food environments. *Listeria monocytogenes*, which is one of pathogens, population growth and inactivation data under 1,007 environmental conditions, including five food categories (beef, culture medium, pork, seafood, and vegetables) and temperatures ranging from 0 to 25 °C, were obtained from the ComBase database (www.combase.cc). We used eXtreme gradient boosting tree, a machine learning algorithm, to predict bacterial population behavior from eight explanatory variables: ‘time’, ‘temperature’, ‘pH’, ‘water activity’, ‘initial cell counts’, ‘whether the viable count is initial cell number’, and two types of categories regarding food. The root mean square error of the observed and predicted values was approximately 1.0 log CFU regardless of food category, and this suggests the possibility of predicting viable bacterial counts in various foods. The data mining approach examined here will enable the prediction of bacterial population behavior in food by identifying hidden patterns within a large amount of data.

## Introduction

Research in food microbiology has led to the accumulation of a large amount of data on bacterial responses to various environments, such as changes in number of bacterial population over time^1^. In predictive microbiology, statistical models are employed to quantitatively evaluate the relationship between growth or inactivation behavior of pathogenic/spoilage bacteria in food and environment^2^. Statistical models in predictive food microbiology can be used to evaluate the effects of processing and storage conditions on the final pathogen contamination level of products^3^. Predictive microbiology is defined as observations of the effects of environmental factors, integration of the data into statistical models, and predictions of bacterial behavior in food^4^. To date, various statistical models have been developed, such as the sigmoid growth functions for growth kinetics^2^ and the log-linear^5^ and Weibull models^6^ for inactivation kinetics to predict bacterial population behavior. In general, datasets employed for statistical and model development are collected with a specific purpose^7^. For example, in the case of bacterial behavior, data are collected to observe inactivation or growth behavior. Thus, most statistical models developed in predictive microbiology focus separately on either microbial growth or inactivation^8^. Furthermore, most predictive models are developed based on the data obtained in laboratory media, and to confirm the accuracy of the model, validation is performed using real food matrices^9^. In other words, various mathematical models for predicting bacterial behavior have been independently developed for distinct experimental conditions.

The food environment can be complex, and quantifying some of its features and their effects on microbial population dynamics may be difficult^10^. This is mainly due to the poor understanding of the combined effects of environmental factors on the function of bacterial growth and inactivation. Specifically, identifying the relationship between bacterial population behavior and multidimensional variables such as temperature, a_w_, pH, and food name is a difficult task. In particular, categorical data such as food names make it more difficult to recognize relationships than numerical data. It becomes difficult to express the relationships between bacterial behavior and the effects of environmental conditions, including categorical data, using statistical models. This is because it is not possible to perform arithmetic operations for categorical data, which are qualitative values^11^. Furthermore, statistical models face difficulties when the number of experimental conditions increases. Therefore, an alternative approach is required that can overcome the problems associated with an increase in the amount of data and can predict bacterial behavior without prior information such as the relationship between bacterial behavior and explanatory variables such as types of food and environmental conditions.

Data mining combines statistical analysis, machine learning, and databases to extract hidden patterns from databases. The core of data mining is machine learning^12^, and various machine learning algorithms have been developed^13^. The relationships between the response and function can be determined empirically from data using machine learning. Statistical models generally require analysts to specify the functional form between the predictor and response variables^14^. This approach requires sufficient knowledge for analysts to specify the appropriate model, such as the relationship between explanatory and objective variables^14^. When analysts do not know the relationship between explanatory variables and objective variables, the misuse of statistical models can lead to prediction errors^15^. However, data mining does not face this issue because the relationship between the predictor and response variables are recognized as a pattern by machine learning and can be specified without the user’s specifications^14^. Data mining has been employed in various fields such as agriculture^13^, ecology^14^ and medicine^16^. Cortez et al. (2009) predicted the taste preference of wine using physicochemical data, such as citric acid, pH, and alcohol. To date, data mining has not been employed in predictive microbiology, even though a large amount of data related to population behavior has been obtained and aggregated. The ComBase database (http://www.combase.cc) has been developed as a means of providing easy access to records of bacterial population behavior obtained in research establishments and publications^1^, and has registered approximately 60,000 records to date. The ComBase database provides bacterial population behavior categorized using various environmental conditions such as temperature, pH, and a_w_, food categories like pork and seafood, and food names such as ham and smoked salmon. By introducing data mining, bacterial population behavior can be predicted from environmental conditions using the large amount of accumulated data. Developing models that predict bacterial behavior based on a large amount of data will lead to objective prediction because a stable prediction would be made regardless of the predictor’s previous experience and knowledge.

In the present study, a data-mining approach was introduced as a proof of concept to predict bacterial population behavior in various foods by using effectively a large amount of data accumulated so far. The Data regarding the change in viable cell number over time of *Listeria monocytogenes* were used as a model study. *L. monocytogenes* is one of the pathogens that cause food poisoning all over the world^17^, and a large amount of data are available. Data for microbial responses to the food environment were collected from the ComBase database and the literature. The collected data included population behavior based on five food categories—‘beef’, ‘culture medium’, ‘pork’, ‘seafood’, and ‘vegetables’—with temperature ranging from 0 °C to 25 °C. The eXtreme gradient boosting tree (XGBoost), a machine learning algorithm that easily handles missing value, was used to predict viable cell counts in both the ComBase database and the literature. The data mining approach would enable the prediction of bacterial population behavior in food by identifying hidden patterns within a large amount of data.

## Results

### Model development and evaluation of model accuracy for the ComBase dataset

**Figure 1:**
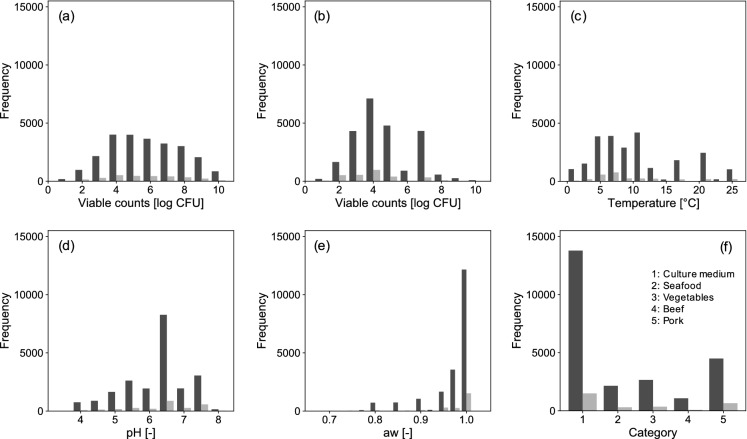
Histograms of the number of observed points for each variable (viable counts (**a**), initial cell numbers (**b**), temperature (**c**), pH (**d**), water activity (**e**), and food category (**f**)). The black and gray bars show the number of training data and test data, respectively.

**Figure 2:**
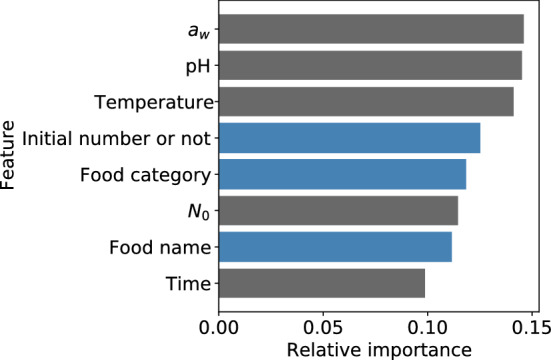
Feature importance of the developed XGBoost model. The X-axis indicates the relative importance, and the Y-axis indicates the explanatory variable name. Blue bars indicate categorical data, and gray ones indicate numerical data.

Figure 1 shows the number of observed points for the training and test data for each feature (viable cell counts, temperature, pH, a_w_, initial cell number, and food category). The data obtained from ComBase were evenly divided into training and test datasets. Figure 2 shows the relative feature importance of the developed XGBoost model. The relative importance of each feature represents the ratio of the importance of each feature when the sum of all feature importance values was 1. All features contributed to model development. Environmental conditions such as a_w_, temperature, and pH contributed the most to model development and to the same extent. Information regarding food such as food category and food name also contributed to some extent to model development.

**Figure 3:**
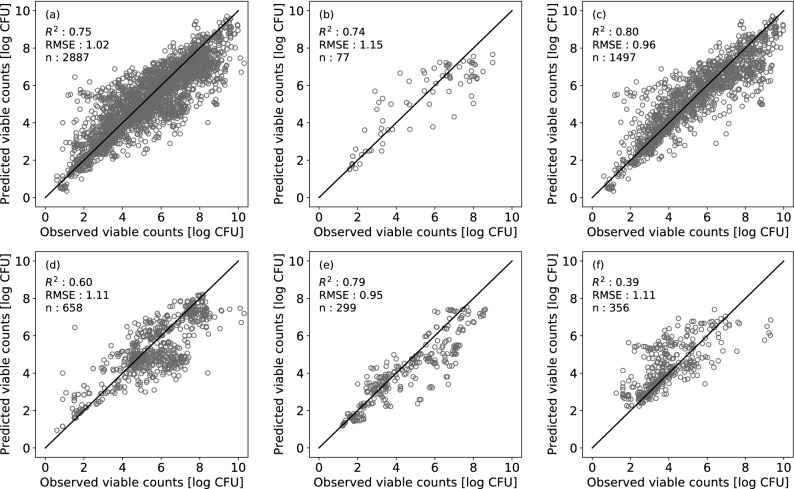
Comparison the observed and predicted values for test data of all food categories (**a**), beef (**b**), culture medium (**c**), pork (**d**), seafood (**e**), and vegetables (**f**). The solid line represents residuals (r) = 0 (log CFU).

**Figure 4:**
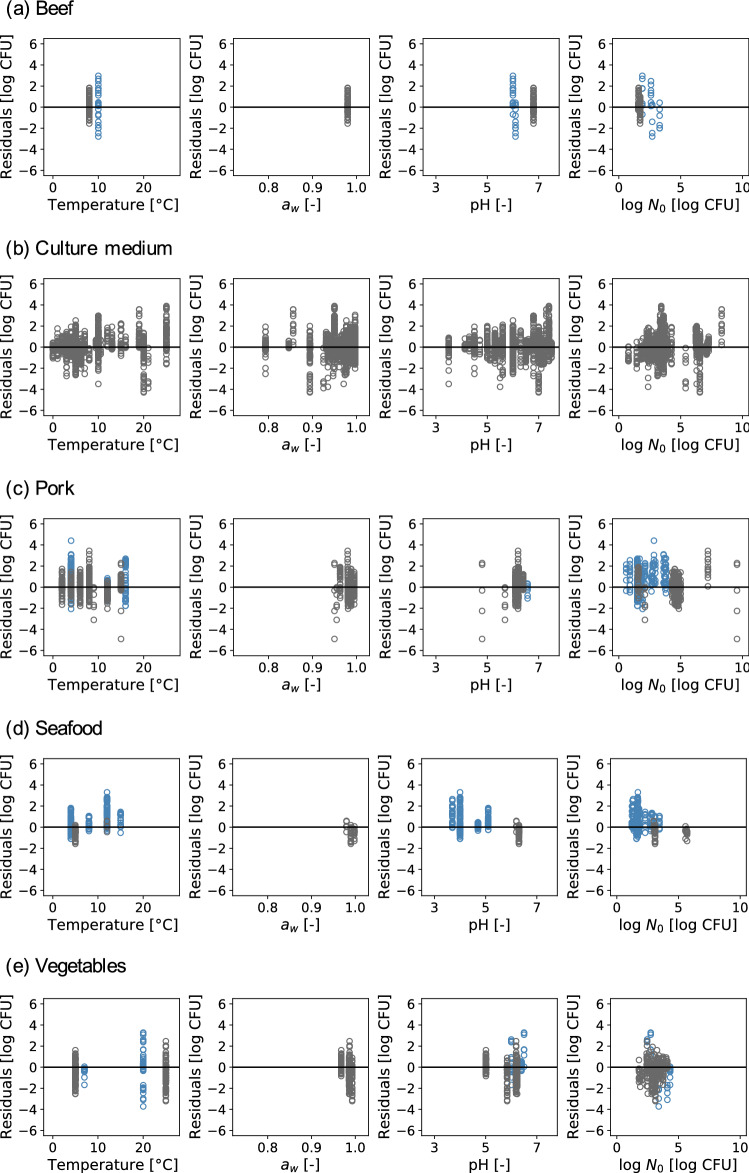
Residual plots of the number of cells predicted for test datasets as functions of temperature, a_w_, pH, and initial cell numbers for beef (**a**), culture medium (**b**), pork (**c**), seafood (**d**), and vegetables (**e**). The blue plots denote data lacking pH and/or a_w_, and the black ones denote data without missing values. The solid line represents residuals (r) = 0 (log CFU).

The number of environmental conditions used in the test dataset was 103, and the number of observed plots was 2,887. Bacterial counts in all test data from ComBase were predicted and plotted by food category against the observed counts (Fig. 3), and the R^2^ and RMSE values were 0.75 and 1.02, respectively. Bacterial counts in the test data from ComBase were also predicted and plotted against the observed counts by food category (Fig. 3). For each food category, the R^2^ values were 0.74, 0.80, 0.60, 0.79, and 0.39 for beef, culture medium, pork, seafood, and vegetables, respectively. The RMSE values for beef, culture medium, pork, seafood, and vegetables were 1.15, 0.96, 1.11, 0.95, and 1.11, respectively. To quantify the model performance, B_f_ and A_f_ were calculated for each food category. The B_f_ values for beef, culture medium, pork, seafood, and vegetables were 0.98, 0.99, 0.91, 0.82, and 1.30, respectively. A bias factor of less than 1 means underestimation, and a bias factor of 1 or more means overestimation. B_f_ > 1 means fail-safe^18^. Other than vegetables, the predicted results were underestimation or close to 1, which was not a big underestimation. The A_f_ values for beef, culture medium, pork, seafood, and vegetables were 1.47, 1.37, 1.46, 1.43, and 1.59, respectively. Furthermore, the residuals were plotted by food category as functions of temperature, a_w_, pH, and initial cell numbers (Fig. 4). Although environmental conditions lacking in pH and/or a_w_ were mixed in all food categories except for the culture medium, residuals were not affected by the presence/absence of missing values (Fig. 4).

### Prediction of bacterial behavior in literature data

**Figure 5:**
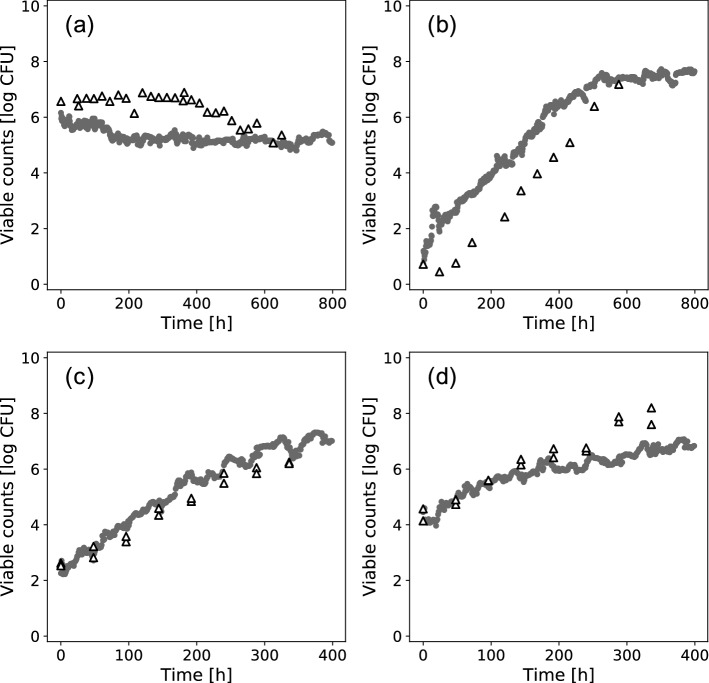
Comparison between the observed and predicted behavior of *Listeria monocytogenes* in culture medium with pH 4.5 and a_w_ 0.997 at 5 °C (**a**), in culture medium with pH 7.3 and a_w_ 0.997 at 4 °C (**b**), and in tuna at 6 °C with initial cell numbers of 2.6 (**c**) and 4.3 (log CFU) (**d**). Predicted results are plotted as a circle. The observed results are plotted as a triangle.

To confirm the applicability of developed model, the developed model was verified using data which were not registered in ComBase. Figure 5a shows the predicted bacterial behavior in TSB (culture medium) at 5 °C, pH 4.5, a_w_ = 0.997, and an initial cell number of 6.6 (log CFU). Similar to the observed value, a slight inactivation behavior was predicted. The developed model could predict bacterial behavior to some extent, with an RMSE of 1.13. Figure 5b shows the predicted bacterial behavior in TSB (culture medium) at 4 °C, pH 7.3, a_w_ = 0.997, and an initial cell number of 0.7 (log CFU). Growth behavior was predicted based on the observed value. The developed model could roughly predict changes in viable cell counts over time with an RMSE of 1.51. Depending on the conditions such as small initial cell numbers, the prediction accuracy of bacterial behavior may be inaccurate (Fig. 5b), because the amount of data used for model development was small. Furthermore, the bacterial behavior in food was also predicted in tuna (seafood) at 6 °C with initial cell numbers of 2.6 (c) and 4.3 (log CFU) (d). The predicted results were compared with the results observed for the three strains. Figure 5c shows the predicted bacterial behavior in tuna with the initial cell numbers of 2.6 (log CFU). When the predicted results were compared with observed values of the three strains, the prediction was performed with high accuracy with RMSE values of 0.99, 0.57, and 0.61. Figure 5d shows the predicted bacterial behavior in tuna with an initial cell number of 4.3 (log CFU). When the predicted results were compared with data for three strains, the RMSE was 1.02, 0.82, and 0.72, and prediction was performed with high accuracy. The growth behavior was predicted under initial cell numbers of 2.6 and 4.3 (log CFU). In condition (c), viable cell counts were predicted under conditions in which the pH and a_w_ were missing. However, even if there were missing values, viable cell counts in foods could be predicted. Predicted inactivation and growth behavior in various food and conditions in the literature could be predicted with RMSE values of approximately 1 (Fig. 5) and with the same accuracy as prediction by test data (Fig. 3).

## Discussion

In the present study, we demonstrated the possibility of applying machine learning to predict bacterial population behavior through a data mining approach using data from ComBase. Categorical data such as food category and food name also contributed to the construction of the model to some extent (Fig. 2). The viable cell counts in food could be roughly predicted in the ComBase database (Fig. 3) and in the literatures (Fig. 5) and the missing value doesn’t affect prediction accuracy (Fig. 4). The data mining approach allowed us to model the complex relationship between food and bacterial population behavior. Although there is still room for improvement in terms of the prediction accuracy, we demonstrated that the accumulated data in a database could be useful for predicting bacterial population behavior through a data mining approach.

One of the roles of ComBase in predictive microbiology is to provide a lot of data. McMeekin (2006) pointed out the possibility of using the discrete data on ComBase for estimating bacterial behavior within interpolation region^10^. Le Marc et al. (2005) used the distinct data on ComBase to develop several models of the growth boundary for each pathogen in culture media^19^. In contrast, no study has been conducted to predict the number of bacteria in food using data of various food using one model. In the present study, a data mining method was introduced as a proof of concept to predict the behavior of bacterial populations from the large amount of distinct data. This data mining approach can be a step toward effective use of data points in database to fast look bacterial population behavior within interpolation region.

Data mining was performed using a large amount of data collected from ComBase and machine learning, and bacterial behavior was predicted for some food category (“culture medium” and “seafood”). In general, the collected data comprise both numerical and categorical variables^11^. Categorical variables represent qualitative attributes and cannot be treated using numerical variables^11^. Therefore, food category and food name were replaced with numerical values as dummy variables to perform machine learning. This is a common technique in models based on decision trees, such as GBDT. Thus, from the feature importance of the model developed, all eight explanatory variables contributed to model development (Fig. 2). Although numerical variables like temperature, pH, and a_w_ contributed the most, because food category and food name also affected model development to some extent, categorical data such as food category and food name would play an important role in model development.

A few previous studies have developed statistical models that encompass quantitative and qualitative information^20–22^. For example, Zwietering et al. (1992) combined qualitative and quantitative information to predict the probability of microbial growth in food. Zwietering et al. (1992) used the physical similarity of food products and compounds contained in a specific product, such as pH, a_w_, and temperature. In statistical modeling, it takes some efforts to select manually the function expressing relationship between the response and explanatory variables. In particular, manually identifying the interaction effects between explanatory variables with high-dimensional data is a difficult task. In contrast, the relationships between the response and explanatory variables can be determined empirically from the data using machine learning through data mining. Even when the number of environmental conditions and the range of environmental conditions increase, the pattern of population behavior is empirically determined using a machine learning algorithm. Thus, a data mining procedure with a machine learning approach can overcome the effects caused by an increase in the number of complicated datasets of population behavior in the food environment. Our model shows an example of the applicability of a data mining approach to a microbial database instead of a statistical approach.

Recently, artificial neural networks (ANNs) have been introduced as a means of modeling the relationship between multiple environmental factors and bacterial responses in the field of predictive microbiology. ANN can reveal knowledge beyond the given information by directly processing the experimental data^23^. ANNs have been employed in analysis of various bacteria and foods^24–28^. Previous studies have not been conducted using a large amount of data taken from the database. As with the ANN introduced in previous studies, XGBoost could predict the number of bacteria as an objective variable from multiple explanatory variables (time, initial cell numbers, temperature, pH, a_w_, food category, and food name) in the present study. A disadvantage of ANN is that it is difficult to explain the relationship between the explanatory variables and the objective variable^23,24^. Thus, quantifying which explanatory variables are important for a predictor is difficult^29^. In contrast, decision trees are suitable for quantifying the importance of features^29^. The XGBoost model is based on decision trees, and the variables that contributed to model development could be identified by visualizing the feature importance (Fig. 2). Recognizing the contribution of variables to model development could help interpret the model.

By introducing data mining using many viable cell counts accumulated in ComBase, we predicted the population behavior of *L. monocytogenes* in the food environment. The bacterial population behavior predicted by this procedure could provide guidelines for determining food processing and storage conditions. Databases that contain information on bacterial behavior and pathogen characteristics play an important role in food safety management^10^. The advantage of using ComBase was the free accessibility. Thus, anyone can perform data mining using machine learning. The data used in the present study were only part of the data registered in ComBase. By applying the procedure introduced in this study to pathogens other than *L. monocytogenes*, bacterial population behavior can be predicted regardless of the type of food category, environmental conditions, and type of bacteria.

## Materials and methods

### Data sets

#### Data selection from ComBase database

The ComBase database contains quantified microbial responses to the food environment with approximately 60,000 records, which have been collated from various research establishments and publications. The data in ComBase include ‘Record ID’, ‘Organism’, ‘Food Category’, ‘Food Name’, ‘Temperature’, ‘pH’, ‘Water activity (a_w_)’, ‘Conditions’, ‘time’ and ‘viable cell counts’. Each dataset of changes in population is assigned a ‘Record ID’, which allows us to recognize one series of experiments of population behavior.

Changes in the population of *Listeria monocytogenes* obtained from the ComBase database were used in this study. Five types of food categories were included because of the large amount of data: ‘beef’, ‘culture medium’, ‘pork’, ‘seafood/fish’, and ‘vegetable or fruit and their product’. In addition, ‘seafood/fish’ and ‘vegetable or fruit and their product’ were abbreviated as ‘seafood’ and ‘vegetables’, respectively. The data used for model development and evaluation were those with temperatures ranging from 0 °C to 25 °C and containing greater than or equal to five observed values in each series of experiments on bacterial population behavior. *L. monocytogenes* can grow in a wide range of temperature (0 to 45 °C)^30^, and ready-to-eat foods that are usually stored at refrigeration temperature are associated with food poisoning due to *L. monocytogenes*^17^, thus the range of lower temperature was selected. In addition, records for which viable counts at 0 h were not present were excluded because the initial cell numbers could not be determined. However, records lacking pH and a_w_ values were included for model development and evaluation. Some records lacked pH and/or a_w_ values in food categories other than culture medium. In particular, records with lacking pH values were also lacking in a_w_ data. The a_w_ of all records for which a_w_ information was not missing in the beef category was 0.98. The number of environmental conditions missing both a_w_ and pH was 92. In total, 2,531 records of bacterial population behavior were extracted for five food categories available in ComBase, and 27,059 viable count data were used. The extracted data from ComBase are summarized in Table 1 by food category. The entire list of “Record ID” and “Food Name” obtained from ComBase can be found as Supplementary Data S1 and Supplementary Data S2 online, respectively.

**Table 1:** Summary of the extracted data from ComBase.

Food category	Temperature	pH	a_w_	^a^Number of food name	Number of viable cell count data		
					Total	^b^Number of missing values	
						a_w_	pH
Beef	3–21	5.5–6.8	0.98	12	1,156	908	681
Culture medium	0–25	3.5–7.5	0.793–0.999	19	15,281	0	0
Pork	0–20	4.8–6.72	0.95–0.998	22	5,155	990	799
Seafood	0–25	3.7–7.2	0.955–0.998	24	2,452	1,606	889
Vegetables	3–25	4.3–7.1	0.750–0.993	35	3,015	1,176	887

^a^Number of Food Name: number of specific food name.

(The entire list of Food Name can be found as Supplementary Data S2 online).

^b^Number of Missing values: number of data lacking a_w_ or pH.

#### Datasets from literature

To confirm the applicability of developed model in general, the bacterial population behavior uncontained in ComBase database were predicted. The literature for external validation was selected, considering that the data in literature was unregistered in ComBase and the environmental conditions can be simply explained using eight explanatory variable. Bacterial cell numbers at a certain time were predicted under three conditions: (a), (b), and (c), which have already been published. The viable cell counts of *L. monocytogenes* in culture medium with pH 4.5 and a_w_ 0.997 at 5 °C was reported by Tiganitas et al.^31^ (a). The viable cell counts of *L. monocytogenes* in culture medium with pH 7.3 and a_w_ 0.997 at 4 °C was reported by Pal et al.^17^ (b). The pH and a_w_ were determined to be common values for TSB, 7.3 and 0.997, respectively, because the culture medium was TSB. Furthermore, viable cell counts of *L. monocytogenes* in tuna at 6 °C was reported by Liu et al.^32^ (c). Because pH and a_w_ were not described clearly, they were treated as missing values.

### Data pre-processing

The data obtained in Sect. 4.1.1 were mixed numerical data and categorical data. For each Record ID, the objective variable was the number of bacteria (log CFU). Eight types of explanatory variables were included: ‘Time (h)’, ‘Temperature (°C)’, ‘pH’, ‘a_w_’, ‘Initial cell number (log CFU)’, ‘Initial number or not’, ‘Food category’, and ‘Food name’. ‘Time’, ‘Temperature’, ‘pH’, ‘a_w_’, and ‘Initial cell number’ were numerical data, which were used without changes for model development. The viable counts at 0 h were used as the initial cell numbers for each record ID. Data with a time of 0 (h) were labeled as 0, and other data were labeled as 1 to characterize whether each record contained the data relevant to the initial cell number. Furthermore, because food category and food name were categorical data, they were converted into numerical values. The five food categories were converted to 0–4, while the 112 different food names were converted to 0–111. The data acquired from ComBase included ‘Record ID’ and could be employed for each series of experimental results of pathogen survival registered based on the record ID. Here, we renamed ‘Record ID’ as ‘Environmental ID’ to avoid overlapping with the environmental conditions in the training and test datasets as follows. The record IDs for which temperature, pH, a_w_, food category, and food name were completely the same were regarded as the results of experiments conducted through different repetitions under the same conditions, and the same ‘Environmental ID’ was reassigned as the result of a single experimental condition. Thus, 2,531 types of record IDs were assigned to 1,007 types of environmental IDs. A part of the dataset obtained from the above procedure is presented in Table 2. Both ComBase and literature data were preprocessed as described above. All preprocessing steps, model development, and statistical analyses were performed in Python (Version 3. 7. 9).

**Table 2:** Sample of product characteristics and storage conditions for the collected dataset.

^a^Index	^b^Environmental ID	Response variable	Explanatory variable							
		Numerical data						Categorical data		
		^c^logN (log CFU)	^d^Time (h)	^e^logN_0_ (log CFU)	Temperature (°C)	a_w_	pH	^f^Food category	^g^Food name	^h^Initial number or not
0	0	6.49	0	6.49	0	0.894	3.5	0	0	0
1	0	5.04	24	6.49	0	0.894	3.5	0	0	1
2	0	4.4	48	6.49	0	0.894	3.5	0	0	1
3	0	3.85	72	6.49	0	0.894	3.5	0	0	1
4	0	3.41	96	6.49	0	0.894	3.5	0	0	1
27,054	1006	5.89	144	2.40	9	0.977	6.11	4	110	1
27,055	1006	7.25	216	2.40	9	0.977	6.11	4	110	1
27,056	1006	7.62	288	2.40	9	0.977	6.11	4	110	1
27,057	1006	8.41	384	2.40	9	0.977	6.11	4	110	1
27,058	1006	8.54	480	2.40	9	0.977	6.11	4	110	1

^a^Index: serial number of viable cell count data.

^b^Environmental ID: serial number of environmental condition.

^c^logN: logarithmic of viable cell number.

^d^Time: elapsed time.

^e^logN_0_: logarithmic of initial cell number.

^f^Food Category: number of food category (0: culture medium, 1: seafood, 2: vegetable, 3: beef, 4: pork).

^g^Food Name: number of food name (0–111), which is described in Supplementary Data S2.

^h^Initial number or not: dummy variable showing whether the viable cell number is the initial cell number (0: initial cell number, 1: not initial cell number).

### Model development

#### XGBoost model

The eXtreme Gradient Boosting Tree (XGBoost), which extends the concept of the gradient boosting decision tree (GBDT). GBDT is an iterative decision tree algorithm which includes multiple decision trees^33^. This algorithm is a machine learning method that combines gradient boosting, which is a step-by-step method focused on gradient reduction of the loss function, and a decision tree, which is a machine learning algorithm. Boosting is an ensemble learning method, which can create a high-performance model by combining multiple weak base models. Tree-based ensemble techniques that combine multiple simple decision trees include random forests, gradient boosting machines, and boosting regression trees. GBDT uses a decision tree as the base model, and gradient boosting trains it sequentially by adding each base model and fixing the errors generated by the previous tree model. The GBDT method has been widely employed in machine learning and data mining studies^34,35^. XGBoost was used in the present study because it can handle missing values without specific processing. XGBoost models were built using the XGBoost Python Package (https://xgboost.readthedocs.io/en/latest/python/index.html).

#### Modelling procedure

**Figure 6:**
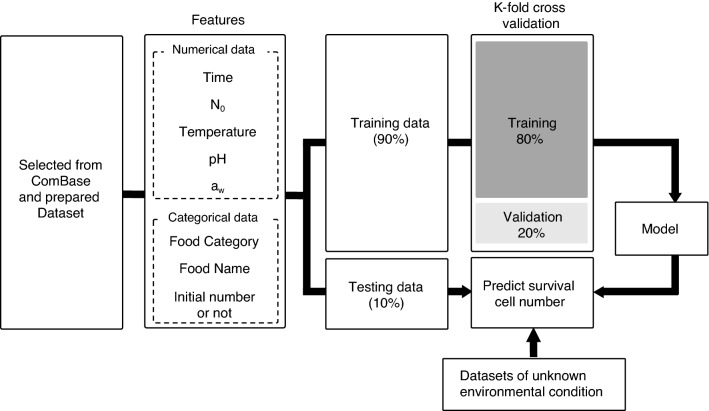
Flow of model development and validation.

We aimed to develop a machine learning model for predicting bacterial responses to food environments characterized by controlling factors such as temperature, pH, and a_w_. The flow of the machine learning process is shown in Fig. 6. First, the dataset was divided into training and testing data. The data included 1,007 types of environmental IDs, and each of the five food categories was split into training data and test data randomly so that the proportion of the number of environmental conditions in the training and test datasets was 9:1. Eight input variables that included five numerical data types—temperature (°C), pH, a_w_, time (h), and initial cell number (log CFU)—and three categorical data types—food category, food name, and initial number or not—were used to develop a model to predict viable counts. Parameters of the XGBoost model used in this study were determined by grid search and a fivefold cross-validation. The XGBoost model parameters were the maximum depth of a tree of 9, min_child_weight of 1, gamma of 0.4, subsample of 0.6, colsample_bytree of 0.65, and learning rate of 0.01.

To interpret the developed model, the importance of each variable was calculated. The importance of the features was evaluated using gain, which is an index showing how much the evaluation criteria could be improved, and calculated by using package (https://xgboost.readthedocs.io/en/latest/python/python_api.html). Feature importance allows us to understand how each explanatory variable contributes to the predicted performance during training of the XGBoost algorithm^36^.

### Evaluation of performance of model

The prediction accuracy of the developed model was evaluated using 103 test datasets that were not used in the training. The coefficient of determination (R^2^) and root mean square error (RMSE) were calculated for all test data and for each food category as an index to evaluate the accuracy of the model. The bias (B_f_) and accuracy (A_f_) factors proposed by Ross^18^ are widely used methods for evaluating model performance in predictive microbiology^37^. B_f_ and A_f_ factors are also used in predictive model of viable counts^38^. Bias and accuracy factors are shown in Eqs. (1) and (2), respectively.

1 $$bias\,factor = 10^{{\left( {\mathop \sum \limits_{i = 1}^{n} \log \left( {pd_{i} /ob_{i} } \right)/n} \right)}} ,$$

2 $$accuracy\,factor = 10^{{\left( {\mathop \sum \limits_{i = 1}^{n} \left| {\log \left( {pd_{i} /ob_{i} } \right)} \right|/n} \right)}}$$

where $pd_{i}$ is the value predicted by the model, $ob_{i}$ is the observed value, and $n$ is the number of data used in the calculation. A bias factor of less than 1 indicates underestimation, and a bias factor of 1 or more indicates overestimation. Since B_f_ cancels overestimation and underestimation, A_f_ was also calculated^39^. A_f_ takes a value of 1 or more, and the larger the value, the lower the prediction accuracy^40^. Furthermore, the residuals ($r$) were calculated from the observed and predicted values as follows:

3 $$r_{i} = y_{i} - \hat{y}_{i} ,$$

where $r_{i}$ (log), $y_{i}$, and $\hat{y}_{i}$ are the $i$ th residual (log), $i$ th observed value (log), and $i$ th predicted value (log), respectively. To determine whether temperature, a_w_, pH, and initial cell numbers affected the residuals, the relationships between each variable (temperature, a_w_, pH, and the initial cell numbers) and the residuals were plotted for each food category. In addition, the data shown in the literature were used to predict the changes in viable cell numbers over time, and evaluated by calculating RMSE from the observed and predicted values.

## Supplementary Information

Supplementary Information 1.

Supplementary Information 2.
